# RPN13/ADRM1 inhibitor reverses immunosuppression by myeloid-derived suppressor cells

**DOI:** 10.18632/oncotarget.12095

**Published:** 2016-09-17

**Authors:** Ruey-Shyang Soong, Ravi K. Anchoori, Benjamin Yang, Andrew Yang, Ssu-Hsueh Tseng, Liangmei He, Ya-Chea Tsai, Richard B.S. Roden, Chien-Fu Hung

**Affiliations:** ^1^ Department of Pathology, Johns Hopkins Medical Institutions, Baltimore, MD, United States; ^2^ Department of Obstetrics and Gynecology, Johns Hopkins Medical Institutions, Baltimore, MD, United States; ^3^ Department of Molecular Microbiology and Immunology, Johns Hopkins Medical Institutions, Baltimore, MD, United States; ^4^ Department of Oncology, Johns Hopkins Medical Institutions, Baltimore, MD, United States; ^5^ Department of General Surgery, Chang Gung Memorial Hospital at Keelung, Keelung City, Taiwan; ^6^ Department of Chang Gung University, College of Medicine, Taoyuan, Taiwan

**Keywords:** RPN13, proteasome, immunosuppression, MDSCs, Stat3

## Abstract

Myeloid-derived-suppressor cells (MDSCs) are key mediators of immune suppression in the ovarian tumor microenvironment. Modulation of MDSC function to relieve immunosuppression may enhance the immunologic clearance of tumors. The bis-benzylidine piperidone RA190 binds to the ubiquitin receptor RPN13/ADRM1 on the 19S regulatory particle of the proteasome and directly kills ovarian cancer cells by triggering proteotoxic stress. Here we examine the effect of RA190 treatment on the immunosuppression induced by MDSCs in the tumor microenvironment, specifically on the immunosuppression induced by MDSCs. We show that RA190 reduces the expression of Stat3 and the levels of key immunosuppressive enzymes and cytokines arginase, iNOS, and IL-10 in MDSCs, while boosting expression of the immunostimulatory cytokine IL-12. Furthermore, we show that the RA190-treated MDSCs lost their capacity to suppress CD8+ T cell function. Finally, we show that RA190 treatment of mice bearing syngeneic ovarian tumor elicits potent CD8+ T cell antitumor immune responses and improves tumor control and survival. These data suggest the potential of RA190 for ovarian cancer treatment by both direct killing of tumor cells via proteasome inhibition and relief of MDSC-mediated suppression of CD8 T cell-dependent antitumor immunity elicited by the apoptotic tumor cells.

## INTRODUCTION

The tumor microenvironment is a complex system consisting in addition to the tumor itself, stromal cells, immune cells, etc. and is impacted by a wide variety of immunogenic and immunosuppressive factors. The dynamic interaction between these components forms a delicate balance and any shift in such balance has a significant impact on tumor growth [1]. In many cases, the immunosuppressive components eventually outweigh their immunogenic counterpart, which leads to escape from immunosurveillance and uncontrolled tumor growth and eventually metastasis.

Important among the immunosuppressive actors are myeloid-derived suppressor cells (MDSCs). MDSCs are a family of cells of myeloid origin including in mice Gr-1^+^CD11b^+^Ly6G^+^ cells from the granulocytic lineage and Gr-1^+^CD11b^+^Ly6C^+^ cells of monocytic lineage [2]. MDSCs mediate immunosuppression primarily through the expression of arginase-1 (Arg1) and inducible nitric oxide synthase (iNOS) [3, 4]. Increased activity of Arg1 leads to depletion of arginine, an essential T cell nutrient, resulting in suppressed T cell proliferation. The metabolic activity of iNOS produces nitric oxide (NO), which induces T cell apoptosis and leads to T cell unresponsiveness by altering the structure of the T cell receptor through its nitration [5, 6]. The expansion and function of MDSCs are heavily regulated by signal transducer and activator of transcription 3 (Stat3) [7–10]. Signaling by Stat3 drives the expression of Bcl-xL, c-Myc, cyclin D1, and survivin, promoting proliferation and preventing apoptosis in MDSCs, as well as inhibiting their differentiation into mature myeloid cells [11]. In addition, Stat3 also regulates the expression of various immunosuppressive factors by MDSCs including of Arg1, iNOS, and IL-10. Due to its key role as a regulator of the immunosuppressive properties of MDSCs, Stat3 is a candidate target for therapies aiming to reverse MDSC-mediated immunosuppression.

In addition to directly killing tumor cells, the ability to generate synergistic therapeutic effect by drugs can enhance its effectiveness and reduce the chance for relapse. Studies have shown that durable remission or cure is typically associated with a concomitant CD8+ cytotoxic T cell response in the tumor microenvironment [12, 13]. For example, the potent chemotherapeutic drug cisplatin, which provides direct tumor killing, can also cause dendritic cells to accumulate in the tumor bed and activate tumor-specific CD8+ T cell responses [12]. This finding suggests that the immunologic balance within tumor microenvironment can be altered by cisplatin to favor CD8+ T cell effector activities. In another study, the receptor tyrosine kinase inhibitor sunitinib was shown to inhibit Stat3, leading to down regulation of angiogenic gene expression as well as the reduction of MDSCs and regulatory T cells, resulting in tumor cell apoptosis and growth arrest in renal cell carcinoma [13]. These results suggest that reduced immunosuppression in the tumor microenvironment promotes CD8+ cytotoxic T cell responses. Thus, in addition to direct tumor cell killing, counteracting immunosuppression within the tumor microenvironment has become the key factor in durable treatment responses.

The bis-benzylidine piperidone RA190 is a novel proteasome inhibitor [14]. While the proteasome inhibitors bortezomib and carfilzomib licensed for treatment of relapsed multiple myeloma act by inhibiting the 20S catalytic core, RA190 irreversibly binds to the ubiquitin receptor RPN13/ADRM1 on the 19S regulatory cap of the 26S proteasome. Knockdown of RPN13 has been shown to disrupt proteasome function and triggers the accumulation of polyubiquinated proteins, leading to endoplasmic reticulum (ER) stress [14, 15]. RA190 also induces apoptosis in various cancer cells including bortezomib-resistant multiple myeloma [14]. The initial study of RA190 demonstrated its potential as a cytotoxic chemotherapeutic agent based on therapeutic activity against ovarian cancer xenograft in immunocompromised mice [14], consistent with a role for RPN13 in ovarian cancer cell proliferation, migration and survival [16]. In the current study, we investigate the effect of RA190 on MDSCs, the tumor microenvironment, and immunosuppression in immunocompetent mice.

## RESULTS

### RA190 reduces Stat3 expression in MDSCs *in vitro*

**Figure 1:**
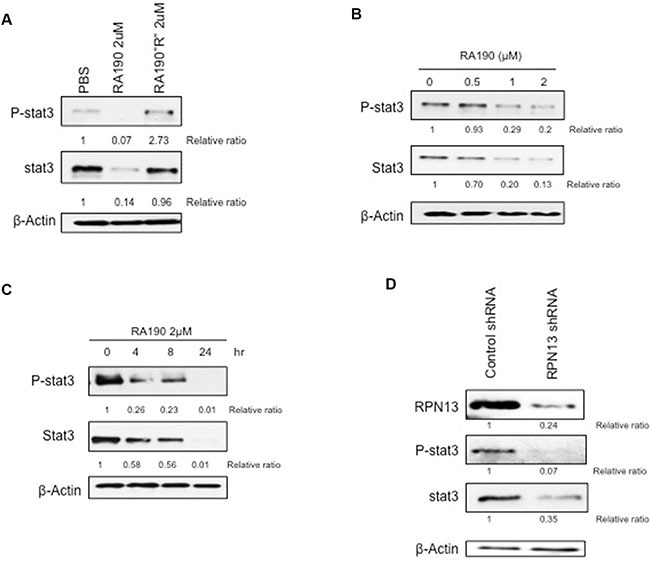
Impact of RA190 treatment or RPN13 knock down on P-Stat3 and Stat3 levels in MDSCs *in vitro*. (**A**) Immunoblot of P-Stat3 and Stat3 expression level of MDSCs treated with PBS, RA190 (2 μM), and the inactive analog RA190R (2 μM). (**B**) Expression of P-Stat3 and Stat3 in MDSCs treated with various doses of RA190 for 8 hours. (**C**) Time course of P-Stat3 and Stat3 expression in MDSCs following RA190 treatment. (**D**) Lentivirus expressing Rpn13 shRNA was used to infect MDSCs and knock down Rpn13 expression. Immunoblot showing Rpn13 protein expression in MDSCs treated with control shRNA and Rpn13 shRNA as well as expression level of P-Stat3 and Stat3.

Human MDSCs express high levels of phosphorylated Stat3 (P-Stat3) [17, 18], but endoplasmic reticulum (ER) stress inhibits Stat3 phosphorylation [19]. RA190, like other proteasome inhibitors, creates profound ER stress by blocking the removal of misfolded proteins. This ER stress triggers the unfolded protein response (UPR) by enhancing IRE1α-mediated splicing of the mRNA coding for the active form of transcription factor XBP1 and elevating the expression of activating transcription factor-4 (ATF-4) and C/EBP-homologous protein (CHOP)-10 [14]. We hypothesized that RA190 induced ER stress would inhibit Stat3 phosphorylation in MDSCs from ovarian tumor bearing mice. We first demonstrated the binding of biotinylated RA190 to a 42kDa cellular protein, a size consistent with RPN13, in MDSCs (Supplementary Figure S1). Then, western blot analysis was used to assess the expression of P-Stat3, Stat3, and ER stress-associated protein ATF4 in MDSCs obtained from the spleens of tumor-bearing mice. We found that the steady state level of P-Stat3 is high in murine MDSCs. However, 8 hours after treatment with 2 μM RA190 the murine MDSC exhibited a profound reduction in the level of both P-Stat3 and Stat3, as determined by relative ratio to PBS-treated control (Figure 1A). Treating MDSCs with the inactive RA190R analog, which lacks proteasome inhibition properties, did not reduce the P-Stat3 or Stat3 levels (Figure 1A). Further more, MDSCs treated with 2 μM RA190 exhibited a significant increase in the level of ATF4 at 8 hours after RA190 treatment compared to the MDSCs that did not receive the treatment (Supplementary Figure S2). This result suggests that inhibition of proteasome function by RA190 is responsible for the reductions of P-Stat3 and Stat3 levels in MDSCs. The reduction of P-Stat3 and Stat3 expression correlates positively with greater RA190 concentration (Figure 1B). Treatment with 2 μM RA190 for 24 hours reduced the P-Stat3 levels to near-background and this concentration was used for subsequent experiments. A reduction in P-Stat3 was apparent after only four hours of RA190 treatment and increased over 24 hours (Figure 1C). In addition, qRT-PCR analysis revealed that MDSCs experienced a significant reduction in Stat3 gene transcription after 24 hours of RA190 treatment as compared to untreated MDSCs (Supplementary Figure S3). These data suggest that the reduction of P-Stat3 in MDSCs upon *in vitro* treatment with RA190 reflects the loss of total Stat3 via reduced Stat-3 mRNA rather than dephosphorylation of P-Stat3 or increased Stat3 turnover.

### MDSCs show reduced IL-10 but increased IL-12 cytokine secretion after treating RA190 *in vitro*

**Figure 2:**
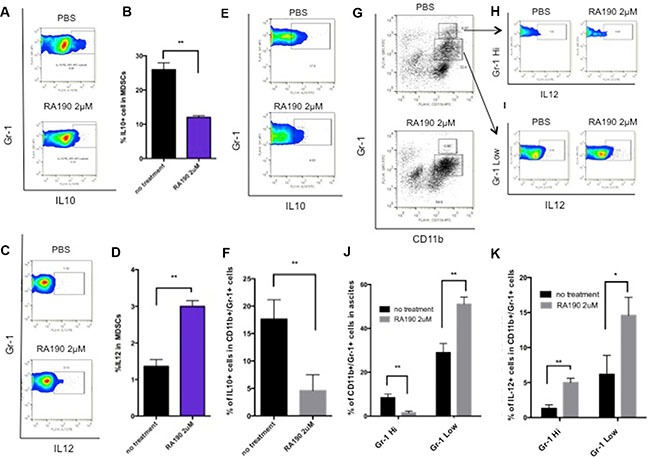
Impact of RA190 treatment upon IL-10, IL-12, and Gr-1 expression in MDSCs isolated from spleen and tumor microenvironment (**A**–**D**) Splenocytes of ID8-Luc tumor bearing mice were treated with or without RA190 (2 μM) for 48 hours and IL-10 and IL-12 expression were assessed in CD11b+ Gr-1+ cells. (A) Representative flow cytometry of IL-10 in MDSCs in spleen. (B) Bar graph showing IL-10 expression in MDSCs in spleen. (C) Representative flow cytometry of IL-12 in MDSCs. (D) Bar graph showing IL-12 expression in MDSCs in spleen. (E-K) Ascites were collected from the peritoneum of ID8-Luc tumor-bearing mice and treated with or without RA190 (2 μM) for 48 hours, and IL-10 and IL-12 expression levels were examined in CD11b+ Gr-1+ cells in the tumor microenvironment. (**E**) Representative flow cytometry of IL-10 in MDSCs in ascites. (**F**) Bar graph showing IL-10 expression level of MDSCs in the ascites. (**G**) MDSC population distribution after PBS or RA190 treatment. (**J**) Bar graph showing the percentage of CD11b+Gr-1^Hi^ cells and CD11b+Gr1^Low^ cells among ascites. (**H**–**I**) Representative flow cytometry analysis of IL-12 in Gr-1^Hi^ and Gr-1^Low^ MDSC populations. (**K**) Bar graph showing IL-12 expression in CD11b+Gr1^Hi^ and CD11b+Gr-1^Low^ populations in various treatment groups. Values are shown as mean ± SD (*P = 0.05, **P = 0.01, ns, not significant).

Next, we examined the changes in MDSC phenotype following treatment with RA190 and the associated reduction of Stat3 levels. IL-10 is one of the main suppressive cytokines secreted by MDSC, and its production is regulated by Stat3 [8]. Splenocytes harvested from ID8-Luc tumor bearing mice were treated with 2 μM RA190 for 8 hours and then analyzed by flow cytometry. The cells were gated for CD11b+/Gr1+ MDSC cell markers and stained with anti-IL10 antibody by intracellular staining. Treatment of these cells with 2 μM RA190 for 8 hours halved their IL-10 expression by MDSCs (Figure 2A–2B). IL-12, a cytokine usually secreted by macrophages, has an immune-potentiating effect within the tumor microenvironment. IL-12 expression was also assessed by intracellular cytokine staining, and interestingly, a doubling of IL-12 production was observed in MDSCs treated with RA190 (Figure 2C–2D).

The cytokine expression profile of MDSCs from ascites collected from the peritoneum of ID8-Luc tumor bearing mice was also examined in the same manner. The ascites cells were treated with 2 μM RA190 for 8 hours and then analyzed by flow cytometry, with gating for CD11b+/GR1+ MDSC cell markers and intracellular staining with anti-IL10 (Figure 2E–2F) or IL12 antibody (Figure 2G–2I). The results obtained for MDSC in the ascites of mice bearing the intra peritoneal ID8-Luc tumor were similar to the cytokine data for MDSC from the spleen. The IL-10 expression level was 3 times higher in untreated MDSCs compared to RA190-treated cells (Figure 2E–2F), whereas IL-12 levels were enhanced by RA190 treatment. Interestingly, a shift in the MDSC population was also observed (Figure 2G–2I). In untreated ascites, about 7% of MDSCs displayed a Gr-1^high^ (G-MDSC) phenotype. However, RA190 treatment caused the majority of MDSCs in ascites to shift to a Gr-1^low^ phenotype (M-MDSC) (Figure 2G). An increase in IL-12 secretion in both MDSC phenotypes was also noted following RA190 treatment (Figure 2H–2K). These results suggest that RA190 treatment is able to both reduce suppressive IL-10 levels and concomitantly increase IL-12 production by MDSCs, which may impact their phenotype and immunosuppressive properties.

### RA190 treatment *in vitro* reduces expression of arginase and iNOS by MDSC

**Figure 3:**
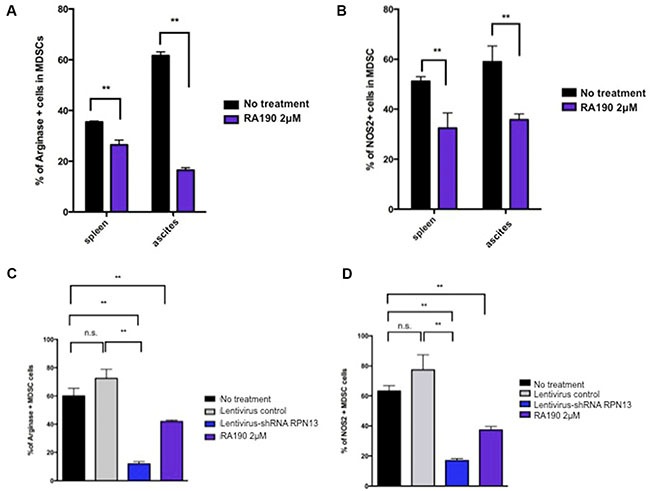
Arginase and iNOS levels in MDSCs isolated from spleen and tumor microenvironment following RA190 treatment or RPN13 knock down *in vitro* (**A** and **B**) MDSCs from spleens and ascites of ID8-Luc tumor-bearing mice were treated with or without RA190 (2 μM) *in vitro* for 24 hours. The levels of Arginase and iNOS were assessed by flow cytometry. (A) Bar graph showing arginase expression in CD11b^+^Gr1^+^ cells isolated from spleen and ascites. (B) Bar graph showing iNOS expression in CD11b^+^Gr1^+^ cells isolation from spleen and ascites. (**C** and **D**) Lentivirus expressing Rpn13 shRNA was used to infect MDSCs and knock down Rpn13 expression. Arginase and iNOS expression in MDSCs receiving no treatment, infected with lentivirus expressing control shRNA, infected with lentivirus expressing Rpn13 shRNA, or treated with RA190 (2 μM) were assessed by flow cytometry. (C) Bar graph showing the percentage of arginase expressing CD11b+Gr-1+ cells in different groups. (D) Bar graph showing the percentage of iNOS expressing CD11b+Gr-1+ cells in different groups. Values are shown as mean ± SD (*P = 0.05, **P = 0.01, ns, not significant).

We performed similar experiments to assess the impact of RA190 on the expression of arginase and iNOS, two immune suppressive factors secreted by MDSCs in the tumor microenvironment. When MDSCs from either splenocytes or ascites harvested from mice bearing intra peritoneal ID8-Luc tumor were treated with 2 μM RA190 *in vitro* for 8 hours, a significant reduction in arginase expression was observed in MDSCs from both spleen and ascites as compared to untreated cells (Figure 3A). A similar reduction in iNOS level was also observed upon exposure of MDSC to RA190 (Figure 3B). These results further imply that RA190 is able to change the phenotype of MDSCs likely by reducing levels of Stat3 and P-Stat3 (Figure 1A–1C), and thus down regulating the production of suppressive molecules such as IL10, arginase and iNOS (Figures 2 and 3A–3B).

### MDSCs treated with RA190 lose the ability to suppress OT-1 T cells *in vitro*

**Figure 4:**
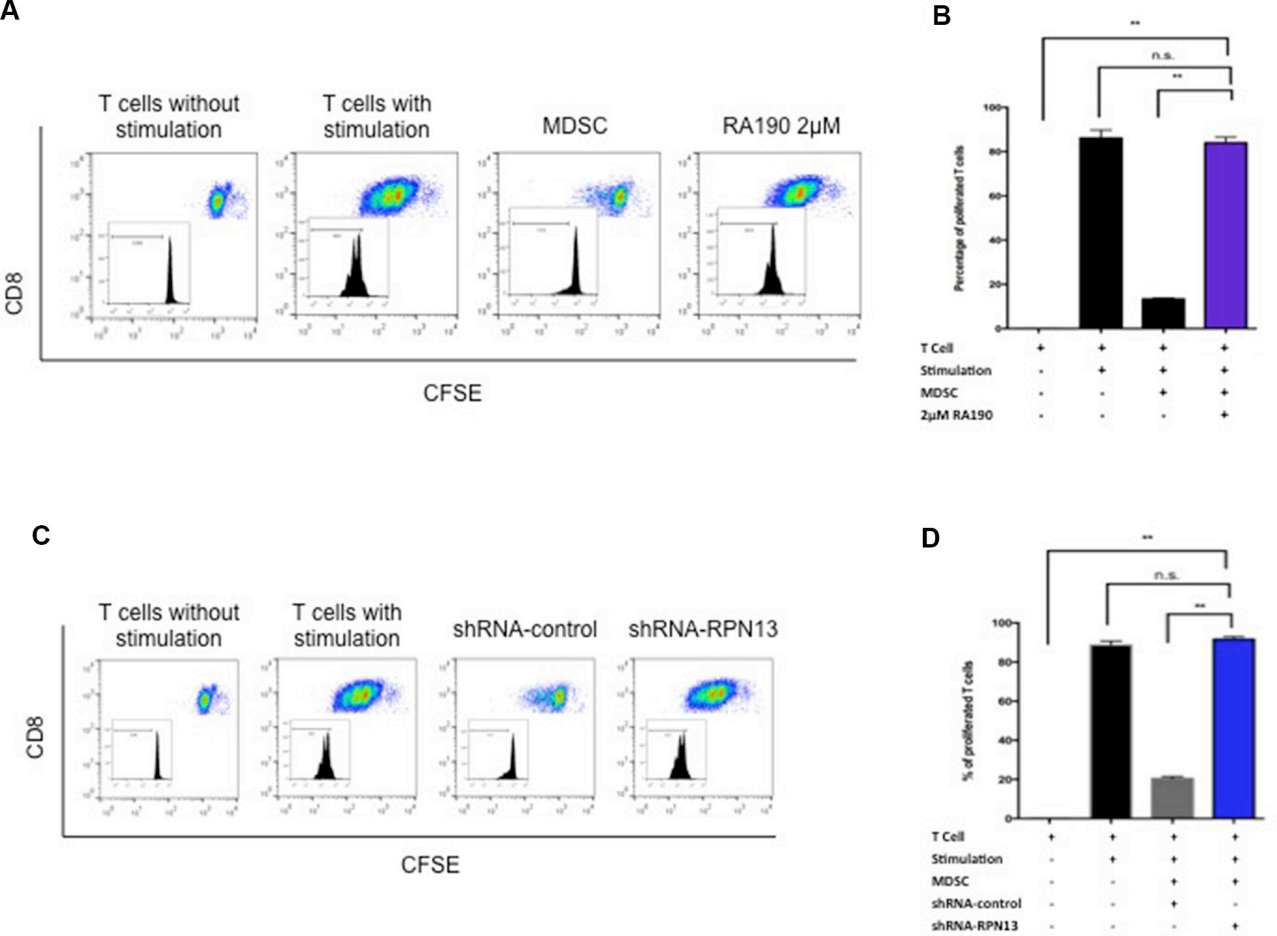
T cell proliferation after co-culturing with MDSCs treated with RA190 or RPN13 knock down *in vitro*. (**A** and **B**) OT-1 T cells stimulated by SIINFEKL peptide loaded on irradiated TC-1 cells were labeled with CFSE, and then co-cultured with RA190-treated MDSCs. (A) Representative flow cytometry of T cell proliferation as measured by CFSE dilution in unstimulated T cells, stimulated T cells, stimulated T cells co-cultured with MDSCs, and stimulated T cells co-cultured with RA190-treated MDSCs. (B) Bar graph depicting the percentage of proliferated OT-1 T cells. (**C** and **D**) OT-1 T cells stimulated by SIINFEKL peptide loaded on irradiated TC-1 cells were labeled with CFSE, and then co-cultured with Rpn13 knocked down MDSCs. (C) Representative flow cytometry of T cell proliferation measured by CFSE dilution in unstimulated T cells, stimulated T cells, stimulated T cells co-cultured with MDSCs infected with control shRNA, and stimulated T cells co-cultured with MDSCs infected with Rpn13 shRNA. (D) Bar graph showing the percentage of proliferated T cells in various treatment groups. Values are shown as mean ± SD (*P = 0.05, **P = 0.01, ns, not significant).

MDSCs exert a strong suppressive effect on T cell function and proliferation in the tumor microenvironment via several mechanisms [8]. We hypothesized that RA190 is able to overcome the suppression effect of MDSCs toward T cells. MDSCs were isolated from splenocytes of ID8-Luc tumor-bearing mice using magnetic beads and then treated with RA190 (2 μM) for 8 hours. OT-1 T cells were labeled with CFSE before stimulation by co-culture with SIINFEKL peptide-loaded irradiated TC-1 tumor cells. 2 × 10^5^ MDSCs and stimulated OT-1 T cells were co-cultured at a 1:1 ratio for three days. On day three, the CFSE signal intensity of OT-1 T cells was analyzed by flow cytometry to assess T cell proliferation via its dilution. A potent suppression of OT-1 cell proliferation was observed when co-cultured with untreated MDSC, as demonstrated by a 9-fold decrease in dilution of CFSE signal intensity compared to stimulated OT-1 cells cultured without MDSCs. However, the CFSE dilution was restored in stimulated OT-1 cells co-cultured with RA190 pre-treated MDSCs, which demonstrated T cell proliferation (Figure 4A–4B). The data indicates that MDSCs pre-treated with RA190 lost their ability to suppress T cell proliferation.

### RPN13 knock down in MDSCs abolishes their T cell suppression function and reduces Stat3 expression

RA190 binds specifically to the Pru domain of the ubiquitin receptor RPN13 that functions in the 19S regulatory particle of the proteasome [14]. Previously, Mazumdar et al. showed knocking down RPN13 in the RAW cell line reduced both NF-κB signaling and iNOS expression [20]. In addition, using an HEK 293 cell-based NF-kB-driven luciferase reporter assay, we confirmed that RA190 decreases NF-kB associated promoter activity in a dose depended fashion (Supplementary Figure S4). Thus, we hypothesized that the ability of RA190 to induce loss of T cell suppression function in MDSC is mediated by RPN13 blockade which interferes with proteasome activities required for NF-κB signaling.

To verify the hypothesis that RA190 exerts its effect through inhibiting the function of RPN13, we first knocked down *Rpn13* in MDSC by transduction with lentivirus carrying ShRNA-*Rpn13*. The *Rpn13* knock down efficiency was confirmed by immunoblot 72 hours after transducing MDSCs with lentivirus expressing either a control shRNA (sh-control) or shRNA-*Rpn13* (sh-*Rpn13*) (Figure 1D). It showed that *Rpn13* knock down by shRNA reduced the rpn13 protein level in MDSCs by 80%. A reduction in P-Stat3 and Stat3 level was also observed in MDSC upon *Rpn13* knock down, as described for MDSCs treated with RA190 (Figure 1). Arginase and iNOS expression levels were also assessed by immunoblot in MDSCs after *Rpn13* knock down. Again, knock down of *Rpn13* in MDSCs triggered a reduction in the levels of both arginase and iNOS (Figure 3C–3D). The *Rpn13* knock down was even more impactful compared to RA190 treatment, producing a 4-fold reduction in arginase level and 2-fold reduction in iNOS, suggesting that RPN13 impacts the expression of Arginase and iNOS. The impact of *Rpn13* knock down on the T cell suppression properties of MDSCs was examined. Peptide-stimulated OT-1 T cells co-cultured with MDSCs treated with control sh-RNA showed reduced proliferation compared to those co-cultured with MDSCs treated with sh-*Rpn13*, as measured by CFSE signal intensity dilution (Figure 4C–4D). This result is consistent with our findings that RA190-treated MDSC lose their capacity to suppress T cell proliferation as compared with untreated MDSC (Figure 4A–4B). These data are consistent with the hypothesis that RA190 abolishes the *in vitro* immunosuppressive function of MDSCs by blocking Rpn13 function, thereby reducing Stat3 levels and lowering production of IL10, Arginase and iNOS.

### Treating mice with RA190 reduces Arginase and iNOS expression in MDSCs, increases the number of CD8+ cytotoxic lymphocytes, and reduces the number of CD4+CD25+FoxP3+ Tregs in the tumor microenvironment

**Figure 5:**
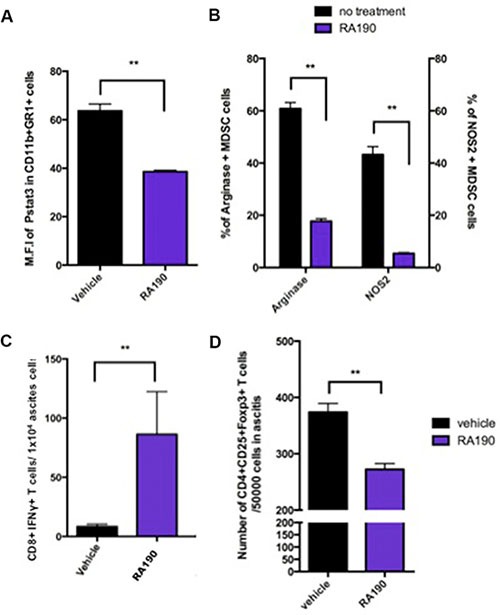
Impact of RA190 treatment upon P-Stat3, arginase and iNOS expression levels in MDSCs, and T cell populations in the tumor microenvironment C57BL/6 mice (*n* = 5/group) were injected with ID8-OVA-Luc tumor cells (2 × 10^6^) intraperitoneally. Mice were treated with RA190 (10 mg/kg/day) beginning on day 11 for five consecutive days and again on day 18 for five consecutive days. (**A**–**B**) MDSCs were isolated from ascites at day23 and analyzed for P-Stat3, arginase, and iNOs expression by intracellular staining and flow cytometry. (A) Bar graph showing mean fluorescent intensity (MFI) of P-Stat3 in the tumor microenvironment of vehicle and RA190-treated groups. (B) Bar graph showing percentage of Arginase^+^ and iNOS^+^ MDSCs in the tumor microenvironment in various groups. (**C**–**D**) Ascites were collected from mice treated with vehicle or RA190 and flow cytometry was used to assess different T cell populations in the tumor microenvironment. (C) Bar graph showing the absolute number of CD8+IFNγ+ T cells in the tumor microenvironment. (D) Bar graph showing the absolute number of CD4+CD25+Foxp3+ T cells in the tumor microenvironment. Values are shown as mean ± SD (*P = 0.05, **P = 0.01, ns, not significant).

We next performed experiments to evaluate the effect of RA190 on MDSCs *in vivo*. C57BL/6 mice (5 mice/group) were injected i.p. with ID8-OVA-Luc tumor cells (2 × 10^6^) and then 11 days later treatment with 10mg/kg RA190 was initiated using the schedule described in Figure 5. On day 23, MDSCs in the peritoneal cavity of vehicle and RA190-treated groups were washed out with PBS, and then assayed by intracellular staining and flow cytometry for P-Stat3, Arginase, and iNOS levels. MDSCs from RA190 treated mice exhibited a reduced level of P-Stat3 (Figure 5A). Furthermore, a 3-fold reduction in both Arginase and iNOS level was observed in MDSCs from RA190 treated mice (Figure 5B). This data is consistent with the *in vitro* findings for RA190-treated MDSCs (Figure 3A–3B). To see whether reduced production of Arginase and iNOs from MDSCs correlated with a greater tumor-specific T cell activity *in vivo*, intracellular cytokine staining and flow cytometry was used to assess IFNy+ CD8+ T cells in the tumor microenvironment. About 9 times more IFNy+ CD8+ T cells were found in the peritoneal washing from RA190-treated mice at 23 days after inoculation with ID8-OVA-Luc tumor cells (Figure 5C). Furthermore, since MDSCs suppress and control immune responses mainly through the generation of Treg in the tumor microenvironment [21, 22], the amount of Tregs in the tumor microenvironment with or without RA190 treatment was also determined. A reduction of Tregs in the tumor microenvironment was observed in mice treated with RA190 as compared to the control animals (Figure 5D). The results show that the RA190 treatment regimen causes alterations in the characteristics of MDSCs and promotes a less immune suppressive tumor microenvironment in mice bearing ID8-OVA-Luc tumor.

### Enhancement of the survival of ID8-OVA-Luc tumor-bearing mice by RA190 treatment requires CD8+ T cells

**Figure 6:**
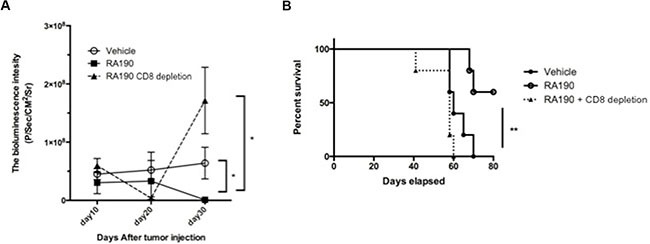
Role of CD8+ T cells in the antitumor effect of RA190 treatment Groups of ID8-OVA-Luc tumor-bearing mice (*n* = 5) were treated with vehicle or RA190 according to the similar schedule described in Figure 5. To deplete CD8+ T cells, a group of mice (*n* = 5) treated with RA190 were also injected intraperitoneally with anti-CD8 monoclonal antibody (100 μg/mouse) on days 11, 12 and 13 after tumor challenge and every other week subsequently. (**A**) Line graph showing tumor growth as measured by bioluminescence intensity in various treatment groups. (**B**) Kaplan–Meier survival analysis of mice in various treatment groups. Values are shown as mean ± SD (*P = 0.05, **P = 0.01, ns, not significant).

Since RA190 treatment *in vitro* reduced the levels of key suppressive molecules produced by MDSC and their capacity to suppress CD8^+^ T cell proliferation, we sought to determine its impact on ovarian tumor control, anti-tumor immunity and the survival of a tumor bearing host. C57BL/6 mice were challenged i.p. with ID8-OVA-Luc tumor cells then treated with vehicle or RA190 as described in Figure 6. ID8-OVA-Luc tumor cells express luciferase that emits bioluminescence upon injection of luciferin substrate which was used to monitor tumor growth every 10 days. Mice treated with RA190 showed a decrease in bioluminescence signal intensity after 20 days and this effect reached significance by day 30 (Figure 6A). Mice treated with vehicle showed a steady growth of signal intensity. This data suggests that RA190 is able to reverse tumor growth *in vivo*. This antitumor effect translates into higher survival in which 60% of RA190 treated mice survived more than 80 days while all of vehicle treated mice died in 70 days (Figure 6B).

We also determined the role of CD8^+^ T cells in the antitumor effect observed in RA190 treated mice by using anti-CD8 antibody to deplete CD8^+^ T cells in one group of mice. CD8^+^ T cell depletion was achieved by injecting anti-mCD8 antibody (100 μg/mouse) intraperitoneally on day 11, 12, 13, and every 7 days thereafter. Mice depleted of CD8^+^ T cells during the treatment with RA190 showed an initial decrease in tumor signal. However, tumor growth increased dramatically by day 30 (Figure 6A). This suggests that RA190 is less able to control tumor growth in the absence of CD8^+^ T cells. The reduction in tumor control by RA190 in CD8 T cell-depleted mice was reflected in poor survival, and all mice depleted of CD8^+^ T cells died in 60 days despite receiving RA190 treatment (Figure 6B). Depletion of CD8^+^ T cells prevented significant control tumor growth or prolongation of survival by RA190, indicating that the antitumor effect elicited by RA190 under this regimen is dependent on the immune activity of CD8^+^ T cells.

## DISCUSSION

Since MDSCs represent a key class of immunosuppressive cells in the tumor microenvironment, numerous drugs have been used to target and modulate their effect [8, 23]. In one of our previous studies, we observed that treatment with cisplatin reduced the MDSC population in TC-1 tumor bearing mice, and was associated with an improved vaccine-mediated antigen-specific antitumor effect [24]. Other chemotherapeutic drugs, such as doxorubicin [25], gemcitabine and 5-fluorouracil also have been shown to inhibit the proliferation and expansion of MDSCs [26]. An alternative approach has been to attempt to attenuate MDSC function or switch their polarization to promote antitumor immunity. All-trans-retinoic acid was shown to induce myeloid cells maturation, effectively lowering the number of MDSCs, which was associated with improved T cell mediated immune responses [27, 28]. Furthermore, vitamin E reduces immunosuppression of MDSCs by neutralizing the reactive oxygen species that they produce [29].

In the current study we found that *in vitro* treatment of MDSCs with RA190 effectively reduces their expression of Stat3 and P-Stat3, which was also associated with lower levels of the immunosuppressive cytokine IL-10, as well as suppressive factors arginase and iNOS. This was observed for both systemic MDSCs harvested from spleen and MDSCs from the tumor microenvironment. Furthermore, RA190 treated MDSCs lost their ability to suppress proliferation of OT-1 T cells *in vitro*. *Rpn13* knock down in MDSCs produced a phenotype similar to RA190 treatment, suggesting that this is the relevant target of RA190 in these cells. This was affirmed by binding of biotinylated RA190 to the expected size protein in MDSC cell lysates. Importantly, either *Rpn13* knock down or RA190 treatment reduced the capacity of MDSC to suppress T cell proliferation *in vitro*. These results, and the increased production of IL12, indicate that RA190 transforms their polarization via binding to Rpn13. This phenotypic switch may reflect the blockade of NF-κB signaling associated with proteasome inhibition, as previously described with the related chalcone curcumin and NF-κB inhibition in TAM [30, 31].

Consistent with our *in vitro* data, RA190 treatment also reduced arginase and iNOS levels in MDSCs in the tumor microenvironment of ID8-luc tumor bearing mice. Greater numbers of IFNγ^+^ CD8^+^ cytotoxic T lymphocytes were also observed in the tumor microenvironment after RA190 treatment. Finally, even though RA190 can directly induce apoptosis of cancer cells (Supplementary Figure S5), we showed that the current RA190 treatment regimen can also reduce ID8 tumor growth and enhance survival in a CD8^+^ T cell-dependent manner. Overall, our results suggest that, in addition to its direct cytotoxic effects on tumor cells, RA190 is able to counteract the immunosuppressive effects of MDSCs, induce a more immunogenic phenotype, and elicit effective CD8^+^ T cell mediated antitumor immunity.

Since RA190 induces cytotoxicity preferentially in various cancer cells [14], it may also produce some degree of cytotoxicity in MDSCs, i.e. the recovery of anti-tumor immunity reflects a loss of MDSC numbers rather than their re-polarization. However, treating MDSCs with 2 μM RA190 did not lead to significant induction of MDSC apoptosis, whereas the ID8 tumor cells were sensitive (Supplementary Figure S5). Indeed, we have previously shown that the high metabolic/UPS stress of ovarian cancer cells renders them more sensitive to proteasome inhibition than non-transformed cells [32]. Thus, the promotion of CD8+ T cell-dependent antitumor immunity by RA190 likely reflects the observed changes in MDSCs phenotype, i.e. re-polarization towards an M1 phenotype, rather than their direct elimination.

Although the major direct effect of proteasome inhibitors is the induction of ER stress, the unfolded protein response (UPR) and eventually apoptosis of cancer cells, several characteristics of RA190 separate it from the licensed proteasome inhibitors bortezomib, iaxomib, marizomib and carfilzomib. All interact with the 20S catalytic particle of the 26S proteasome and disrupt proteasome degradation primarily by directly inhibiting proteolysis [33]. RA190 is the first proteasome inhibitor to interact with Rpn13 in the 19S regulatory particle. Bortezomib differs from RA190 mechanistically, and there is some evidence of synergistic activity which may reflect the inability of bortezomib to inhibit all of the proteolytic 20S activities.

While it is known that RA190 binds covalently to RPN13, how it inhibits proteasomal degradation is less clear. Besche et al demonstrated that RPN13 may undergo ubiquitination under certain conditions such as when exposed to proteasome inhibitors like bortezomib. Further they demonstrated that the ubiquitination of RPN13 prevented it from binding to other ubiquitinated proteins, resulting in its inactivation [34], although it is not clear if this happens in the presence of RA190.

Hamazaki et al demonstrated that even though RPN13 plays an important role in mediating ubiquitinated protein degradation, its function is at least partially complemented by RPN10 and that knockout of RPN13 resulted in a less severe toxic effect than RPN10 [15]. Consistent with this observation, RA190 treatment is less toxic and relatively well tolerated by non-tumor cells such as MDSCs, resulting in mainly phenotypic changes rather than direct cell death (Supplementary Figure S5).

It is not clear how proteasome inhibition, including via RA190, impact antigen presentation. NY-ESO-1 is an important ovarian cancer antigen and Golnik et al observed that NY-ESO-1 antigen presentation is only weakly inhibited by RPN13 knockdown, whereas RPN10 knockdown profoundly inhibited it [35]. This may account for the ability of RA190 to inhibit proteasome function yet still maintain antigen presentation and increase antitumor CD8+ T cell responses (Figure 6B).

Our study indicates that RA190, by reversing the immunosuppressive phenotypes of MDSCs, is able to shift the tumor microenvironment into the direction of being more immunogenic. Interestingly, we reported a similar tumor microenvironment manipulation effect with bortezomib including enhanced the phagocytic activities and maturation of CD11c^+^ dendritic cells *in vitro* [36]. Treatment of mice bearing a murine ovarian tumor, HM-1, with bortezomib induced dendritic cell maturation which correlated with more potent antitumor effects. These results suggest the potential of proteasome inhibitors as a class of chemotherapeutic agents to modulate the tumor microenvironment and enhance CD8^+^ T cell immune response in addition to their direct cytotoxic effects on tumor cells.

Currently, the effectiveness of most cancer immunotherapies is compromised by the immunosuppressive network in the tumor microenvironment. Our data suggest that the novel proteasome inhibitor RA190 can be used to reverse the immunosuppressive phenotype of MDSCs into a more immunogenic profile, and promote activities of CD8^+^ T cells in the tumor microenvironment. Future studies should evaluate the use of RA190 in combination with other immunotherapies.

Rpn13 has been identified as a putative oncogene in ovarian cancer that promotes tumor cell survival, proliferation and metastasis [16]. Indeed, *RPN13* is frequently amplified in human ovarian cancer, although amplification does not appear to correlate with sensitivity to RA190 [16]. These properties suggest RPN13 as a promising target for therapy, and indeed early data suggest a significant cytotoxic effect in human ovarian cancer cells. Ovarian cancer cells exhibit elevated UPS stress, reflecting that higher metabolic rate and protein production. Indeed, they are susceptible to proteasomal inhibition by bortezomib or RA190. However, early clinical trials of bortezomib in ovarian cancer patients were not promising, either alone or in combination with carboplatin, doxorubicin or oxiplatin. Unfortunately, its effect on antitumor immunity and MDSC was not assessed in these studies.

Intraperitoneal RA190 treatment at 10 mg/kg significantly inhibited the growth of human ovarian cancer ES2 xenografts in immunodeficient mice [14]. A comparison of the pharmacokinetics in plasma of RA190 when administered orally or intraperitoneally to mice revealed RA190 was more stable and exhibited a significantly longer half-life in blood circulation when administered intraperitoneally as compared to oral administration [14]. Thus, in the current study in immunocompetent mice, we have again selected 10 mg/kg IP RA190 treatment as the administration dosage and route. We also note that chemotherapy for ovarian cancer is frequently given IP and this route has demonstrated superiority over i.v. administration [37]. This study in immunocompetent animals demonstrates that RA190, in addition to directly eliciting apoptosis in ovarian tumor through ER stress, can also enhance CD8+ T cell-dependent antitumor immunity by attenuating and repolarizing the suppressive functions of MDSCs, an approach that warrants further exploration as a treatment for ovarian cancer.

## MATERIALS AND METHODS

### Animal studies and tumor models

6- to 8-week-old female C57BL/6 mice were purchased from the National Cancer Institute-Frederick Animal Production Area (Frederick, MD). All the mice were housed in the oncology animal facility of the Johns Hopkins Hospital (Baltimore, MD). All animal procedures were performed according to approved protocols and in accordance with recommendations for the proper use and care of laboratory animals.

The ID8-OVA cell line was originally derived from spontaneous transformed mouse ovarian surface epithelial cells, and then transduced to express the ovalbumin gene [38], and was a kind gift from Tahiro Shin of University of Texas Health Science Center in February 2011. The cell line was authenticated and expression of OVA was tested every week by OT1 T cell interferon gamma activation assay. In order to trace the tumor growth *in vivo*, ID8-OVA cells were transduced with a lentivirus construct to stably express luciferase (ID8-OVA-LUC) as previously described [39]. Cells were cultured in RPMI1640 medium containing 10% FBS, 2 mM L-glutamine, 1 mM sodium pyruvate, 2 mM non-essential amino acids, and 50 units/ml streptomycin in a humidified atmosphere of 5% CO_2_/95% air at 37°C.

### RA190 and RA190R

RA190, and its inactive analog RA190R, were synthesized in-house and purified as previously described [14].

### Isolation of MDSCs from ascites and spleen

For ID8-OVA-LUC tumor-bearing mice, ascites were directly aspirated from the peritoneal cavity. To prepare MDSCs from splenocytes, the spleens of mice bearing ID8-OVA-LUC tumor mice were ground through a cell strainer and then red blood cells were lysed in ACK buffer. A myeloid-derived suppressor cell isolation kit (Miltenyi Biotec, San Diego) was used following the manufacturer's protocol and MDSCs at a purity of > 85% were obtained after isolation using magnetic beads.

### Flow cytometry analysis

Antibodies used in the current study were Anti-CD11b PE (eBioscience M1/70)/APC (BD,M1/70), anti-GR-1 FITC (BD RB6-8C5), anti-Ly6G FITC (RB5-8C5) APC (RB5-8C5), anti-Ly6C PreCP-Cr™5.5 (BD AL-21), anti-IL10 FITC (BD), anti-IL12 (P40/P70) (BD), anti-p-Stat3 PE (BD PY705), anti-NOS2 PE (eBioscience CXNFT), arginase FITC (R&D), anti-CD8 PE (BD), anti-IFNγ FITC (BD).

The numbers of IFN-γ-secreting CD8^+^ T cells were analyzed by flow cytometry after adding Golgi plug (1 μg/mL, BD Pharmingen) for 8 hours. Analysis was performed on a Becton-Dickinson FACScan with CELLQuest software (Becton-Dickinson Immunocytometry System, Mountain View, CA) and Flowjo 10 software.

### T cell suppression assay

OVA-specific CD8+ T cell line (OT1) was prepared by harvesting splenocytes from OT-1 transgenic RAG−/− mice and stimulation with irradiated and OVA peptide (SIINFEKL)-pulsed TC-1 cells in the presence of murine IL-2 (20 IU/ml). 1 × 10^5^ irradiated TC-1 cells loaded with SIINFEKL peptide were seeded onto a 96 well plate for 4 hours. 5 × 10^5^ MDSCs isolated from splenocytes of tumor-bearing mice were co-cultured with CFSE (5 mg/mL) labeled OT-1 T cells, previously described [40], at a one to one ratio. On day 3, the CFSE intensity of OT-1 T cells was measured by flow cytometry.

### Western blot analysis

50 μg of protein from the MDSC lysate was denatured using SDS-PAGE and transferred to nitrocellulose membranes (GE Bioscience). After blocking with 5% skim milk in PBS–0.1% Tween 20 (PBST) for 1 hour at room temperature, membranes were incubated overnight with primary antibody at 4°C. Membranes were then washed with PBST and incubated with horseradish peroxidase (HRP)–conjugated secondary antibody before visualization with ECL plus (GE Bioscience). All antibodies, including P-Stat3 (Tyr705, D3A7), Stat3 (cell signaling), b-actin (Santa Cruz), RPN-13 (sigma), and ATF-4 (SC-200; Santa Cruz), were diluted in blocking buffer. The dilution ratio of the antibody was based on manufacturer's recommendation. The Western blot results were quantified by the software ImageJ64 [41], and the relative ratio was compared with the control group.

### Biotin labeling assay

1 × 106 MDSC cells were lysed in 300 μL MPER buffer (Pierce) and the lysate was centrifuged at 13K RPM for 2 min at 4°C to remove cell debris. Lysate supernatant was incubated with Dyna beads streptavidin MyT1 beads (100 μL) for 45 min at 4°C to remove non-specific biotin binding. Beads were removed by a magnet and equal volumes of supernatant were incubated with RA190B for 45 min at 4°C on a plate shaker.

Equal amount of samples were boiled in Laemmli buffer, separated using 4–15% SDS-PAGE, and transferred to a PVDF membrane overnight at 4°C (24 V). The membrane was blocked with 5% BSA in phosphate-buffered saline with Tween-20 for 1 h, washed and probed with HRP-streptavidin (diluted 1:10,000) in PBST for 1 h at room temperature. After washing, the blot was developed using HyGLO chemiluminescent detection reagent (Denville) by BioRad Gel Doc imager.

### Lentiviral transfer and RPN13 knock down by shRNA

RPN13 shRNA (TRC no. TRCN0000125944; sequence, CCGG-CATGCAGAACAATGCCAAAT- CTCGAG-ATTTGGCATTGTTCTGCATGG-TTTTTG) and the nontarget shRNA control vector (cat. no. SHC002; sequence, CCGGCGTGATCTTCACCGACAA GATCTCGAGATCTTGTCGGTGAAGATCACGTTTTT) were purchased from Sigma-Aldrich (St. Louis, MO).

The construct was transfected into 293T cell line, and the virion-containing supernatant was collected 48 hours after transfection. The supernatant was immediately clarified using a 0.45 μM cellulose acetate syringe filter (Nalgene, Rochester, NY) and used to infect MDSCs in the presence of 8 μg/mL polybrene (Sigma, St. Louis, MO) as previously described [42]. One day after lentiviral transfection, the virus supernatant was replaced with normal culture medium. Gene expression level of RPN13 was verified by staining the cell lysates with anti-RPN13 antibody (Sigma) and Western blot analysis.

### q-RT-PCR analysis of STAT3 mRNA expression with SYBR GREEN

RNA was extracted by using TRIzol® Reagent (Invitrogen) according to the manufacturer's instructions. 2 μg RNA was reverse transcription by using SuperScript^®^ III Reverse Transcriptase (Invitrogen). Real-time PCR was analyzed by using CFX Connect™ Real-Time PCR Detection System (Bio-Rad Laboratories) for expression of STAT3 and GAPDH. GAPDH was used as an internal control. The following sense and antisense of STAT3 and GAPDH were: mouse STAT3, 5′–ACCAACATCCTGGTGTCTCC–3′ (sense) and 5′–CATGTCAAACGTGAGCGACT–3′ (antisense) [38]; mouse GAPDH, 5′-GCACAGTCAAGGCCGAGAAT-3′ (sense) and 5′-GCCTTCTCCATGGTGGTGAA-3′ (antisense) [43]. 2 μL cDNA sample was used for PCR amplification with iQ™ SYBR^®^ Green supermix (Bio-Rad Laboratories) according to the manufacturer's protocol.

### Luciferase assay for NF-kB activation

HEK 293 cells transiently transfected with a luciferase reporter construct driven by either an NF-κB-dependent promotor (NF-κB/FL) or a constitutive promoter (Luc) were treated with RA190 or Bortezomib, and TNFα (10 ng/ml) for 7 h. Upon the addition of luciferin, bioluminescence was measured in cell lysates using a luminometer.

### *In vivo* tumor treatment

Five mice per group were challenged intraperitoneally with 3 × 10^5^ ID8-Luc cells per mouse. Three days after tumor challenge, mice were checked by the IVIS system to confirm tumor establishment. The tumor bearing mice were then treated intraperitoneally (i.p.) with 10 mg/kg RA190 formulated in 20% (w/v) 2-hydroxypropyl-β-cyclodextrin in water [14]. Tumor burden was measured by bioluminescence intensity by IVIS system every 7 days. The ID8-OVA-Luc model grows more slowly than ID8-Luc. Therefore 2 × 10^6^ ID8-OVA-Luc tumor cells were injected intraperitoneally and drug treatment was started on day 14. The behavior and body weight of the mice were monitored regularly; upon observation of stress or a change in body weight of more than 20% from average, mice were euthanized based on the animal protocol. Survival statistics were calculated by the log-rank (Mantel-Cox) test using GraphPad Prism 6 software.

### Statistical analysis

All data are expressed as mean ± S.E. where indicated and are representative of at least two separate experiments. Comparisons between individual data points for intracellular cytokine staining with flow cytometric analysis and tumor treatment were made using Student's *t-test*. In the tumor treatment experiments, the principle outcome of interest was the duration of survival until euthanasia based on the animal protocol (in stress, weight change greater than 20%). The event-time distributions for different mice were compared using the Kaplan–Meier method and the log-rank statistic by Prism 6 software. All *p*-values < 0.05 were considered significant.

## SUPPLEMENTARY MATERIALS FIGURES



## References

[1] Pitt JM, Marabelle A, Eggermont A, Soria JC, Kroemer G, Zitvogel L (2016). Targeting the tumor microenvironment: removing obstruction to anticancer immune responses and immunotherapy. Ann Oncol.

[2] Youn JI, Nagaraj S, Collazo M, Gabrilovich DI (2008). Subsets of myeloid-derived suppressor cells in tumor-bearing mice. J Immunol.

[3] Rodriguez PC, Ochoa AC (2008). Arginine regulation by myeloid derived suppressor cells and tolerance in cancer: mechanisms and therapeutic perspectives. Immunological reviews.

[4] Mazzoni A, Bronte V, Visintin A, Spitzer JH, Apolloni E, Serafini P, Zanovello P, Segal DM (2002). Myeloid suppressor lines inhibit T cell responses by an NO-dependent mechanism. J Immunol.

[5] Gallina G, Dolcetti L, Serafini P, De Santo C, Marigo I, Colombo MP, Basso G, Brombacher F, Borrello I, Zanovello P (2006). Tumors induce a subset of inflammatory monocytes with immunosuppressive activity on CD8+ T cells. Journal of Clinical Investigation.

[6] Movahedi K, Guilliams M, Van den Bossche J, Van den Bergh R, Gysemans C, Beschin A, De Baetselier P, Van Ginderachter JA (2008). Identification of discrete tumor-induced myeloid-derived suppressor cell subpopulations with distinct T cell–suppressive activity. Blood.

[7] Kujawski M, Kortylewski M, Lee H, Herrmann A, Kay H, Yu H (2008). Stat3 mediates myeloid cell–dependent tumor angiogenesis in mice. The Journal of clinical investigation.

[8] Gabrilovich DI, Nagaraj S (2009). Myeloid-derived suppressor cells as regulators of the immune system. Nature Reviews Immunology.

[9] Yu H, Pardoll D, Jove R (2009). STATs in cancer inflammation and immunity: a leading role for STAT3. Nature Reviews Cancer.

[10] Mace TA, Ameen Z, Collins A, Wojcik S, Mair M, Young GS, Fuchs JR, Eubank TD, Frankel WL, Bekaii-Saab T, Bloomston M, Lesinski GB (2013). Pancreatic cancer-associated stellate cells promote differentiation of myeloid-derived suppressor cells in a STAT3-dependent manner. Cancer research.

[11] Condamine T, Gabrilovich DI (2011). Molecular mechanisms regulating myeloid-derived suppressor cell differentiation and function. Trends in immunology.

[12] Kang TH, Mao CP, Lee SY, Chen A, Lee JH, Kim TW, Alvarez RD, Roden RB, Pardoll D, Hung CF, Wu TC (2013). Chemotherapy acts as an adjuvant to convert the tumor microenvironment into a highly permissive state for vaccination-induced antitumor immunity. Cancer research.

[13] Xin H, Zhang C, Herrmann A, Du Y, Figlin R, Yu H (2009). Sunitinib inhibition of Stat3 induces renal cell carcinoma tumor cell apoptosis and reduces immunosuppressive cells. Cancer research.

[14] Anchoori Ravi K, Karanam B, Peng S, Wang Joshua W, Jiang R, Tanno T, Orlowski Robert Z, Matsui W, Zhao M, Rudek Michelle A, Hung C-f, Chen X, Walters Kylie J (2013). A bis-Benzylidine Piperidone Targeting Proteasome Ubiquitin Receptor RPN13/ADRM1 as a Therapy for Cancer. Cancer Cell.

[15] Hamazaki J, Hirayama S, Murata S (2015). Redundant Roles of Rpn10 and Rpn13 in Recognition of Ubiquitinated Proteins and Cellular Homeostasis. PLoS Genet.

[16] Fejzo MS, Anderson L, von Euw EM, Kalous O, Avliyakulov NK, Haykinson MJ, Konecny GE, Finn RS, Slamon DJ (2013). Amplification Target ADRM1: Role as an Oncogene and Therapeutic Target for Ovarian Cancer. International journal of molecular sciences.

[17] Vasquez-Dunddel D, Pan F, Zeng Q, Gorbounov M, Albesiano E, Fu J, Blosser RL, Tam AJ, Bruno T, Zhang H (2013). STAT3 regulates arginase-I in myeloid-derived suppressor cells from cancer patients. The Journal of clinical investigation.

[18] Niederreiter L, Fritz TM, Adolph TE, Krismer AM, Offner FA, Tschurtschenthaler M, Flak MB, Hosomi S, Tomczak MF, Kaneider NC, Sarcevic E, Kempster SL, Raine T (2013). ER stress transcription factor Xbp1 suppresses intestinal tumorigenesis and directs intestinal stem cells. The Journal of experimental medicine.

[19] Kimura K, Yamada T, Matsumoto M, Kido Y, Hosooka T, Asahara SI, Matsuda T, Ota T, Watanabe H, Sai Y (2012). Endoplasmic reticulum stress inhibits STAT3-dependent suppression of hepatic gluconeogenesis via dephosphorylation and deacetylation. Diabetes.

[20] Mazumdar T, Gorgun FM, Sha Y, Tyryshkin A, Zeng S, Hartmann-Petersen R, Jorgensen JP, Hendil KB, Eissa NT (2010). Regulation of NF-kappaB activity and inducible nitric oxide synthase by regulatory particle non-ATPase subunit 13 (Rpn13). Proc Natl Acad Sci USA.

[21] Huang B, Pan PY, Li Q, Sato AI, Levy DE, Bromberg J, Divino CM, Chen SH (2006). Gr-1+CD115+ immature myeloid suppressor cells mediate the development of tumor-induced T regulatory cells and T-cell anergy in tumor-bearing host. Cancer research.

[22] Lindau D, Gielen P, Kroesen M, Wesseling P, Adema GJ (2013). The immunosuppressive tumour network: myeloid-derived suppressor cells, regulatory T cells and natural killer T cells. Immunology.

[23] Wesolowski R, Markowitz J, Carson W (2013). Myeloid derived suppressor cells–a new therapeutic target in the treatment of cancer. Journal for immunotherapy of cancer.

[24] Tseng CW, Hung CF, Alvarez RD, Trimble C, Huh WK, Kim D, Chuang CM, Lin CT, Tsai YC, He L, Monie A, Wu TC (2008). Pretreatment with cisplatin enhances E7-specific CD8+ T-Cell-mediated antitumor immunity induced by DNA vaccination. Clinical cancer research.

[25] Alizadeh D, Trad M, Hanke NT, Larmonier CB, Janikashvili N, Bonnotte B, Katsanis E, Larmonier N (2014). Doxorubicin eliminates myeloid-derived suppressor cells and enhances the efficacy of adoptive T-cell transfer in breast cancer. Cancer research.

[26] Talmadge JE, Gabrilovich DI (2013). History of myeloid-derived suppressor cells. Nature Reviews Cancer.

[27] Mirza N, Fishman M, Fricke I, Dunn M, Neuger AM, Frost TJ, Lush RM, Antonia S, Gabrilovich DI (2006). All-trans-retinoic acid improves differentiation of myeloid cells and immune response in cancer patients. Cancer research.

[28] Iclozan C, Antonia S, Chiappori A, Chen DT, Gabrilovich D (2013). Therapeutic regulation of myeloid-derived suppressor cells and immune response to cancer vaccine in patients with extensive stage small cell lung cancer. Cancer immunology, immunotherapy.

[29] Kang TH, Knoff J, Yeh WH, Yang B, Wang C, Kim YS, Kim TW, Wu TC, Hung CF (2014). Treatment of Tumors with Vitamin E Suppresses Myeloid Derived Suppressor Cells and Enhances CD8+ T Cell-Mediated Antitumor Effects. PloS one.

[30] Tu SP, Jin H, Shi JD, Zhu LM, Suo Y, Lu G, Liu A, Wang TC, Yang CS (2012). Curcumin induces the differentiation of myeloid-derived suppressor cells and inhibits their interaction with cancer cells and related tumor growth. Cancer Prev Res (Phila).

[31] Hagemann T, Lawrence T, McNeish I, Charles KA, Kulbe H, Thompson RG, Robinson SC, Balkwill FR (2008). “Re-educating” tumor-associated macrophages by targeting NF-kappaB. The Journal of experimental medicine.

[32] Bazzaro M, Lin Z, Santillan A, Lee MK, Wang MC, Chan KC, Bristow RE, Mazitschek R, Bradner J, Roden RB (2008). Ubiquitin proteasome system stress underlies synergistic killing of ovarian cancer cells by bortezomib and a novel HDAC6 inhibitor. Clinical cancer research.

[33] Bedford L, Lowe J, Dick LR, Mayer RJ, Brownell JE (2010). Ubiquitin-like protein conjugation and the ubiquitin–proteasome system as drug targets. Nature reviews Drug discovery.

[34] Besche HC, Sha Z, Kukushkin NV, Peth A, Hock EM, Kim W, Gygi S, Gutierrez JA, Liao H, Dick L, Goldberg AL (2014). Autoubiquitination of the 26S proteasome on Rpn13 regulates breakdown of ubiquitin conjugates. EMBO J.

[35] Golnik R, Lehmann A, Kloetzel PM, Ebstein F (2016). Major Histocompatibility Complex (MHC) Class I Processing of the NY-ESO-1 Antigen Is Regulated by Rpn10 and Rpn13 Proteins and Immunoproteasomes following Non-lysine Ubiquitination. J Biol Chem.

[36] Chang CL, Hsu YT, Wu CC, Yang YC, Wang C, Wu TC, Hung CF (2012). Immune mechanism of the antitumor effects generated by bortezomib. J Immunol.

[37] Armstrong DK, Fujiwara K, Jelovac D (2012). Intraperitoneal treatment in ovarian cancer: the gynecologic oncology group perspective in 2012. Am Soc Clin Oncol Educ Book.

[38] Tomihara K, Guo M, Shin T, Sun X, Ludwig SM, Brumlik MJ, Zhang B, Curiel TJ, Shin T (2010). Antigen-specific immunity and cross-priming by epithelial ovarian carcinoma-induced CD11b(+)Gr-1(+) cells. J Immunol.

[39] Chang CL, Hsu YT, Wu CC, Lai YZ, Wang C, Yang YC, Wu TC, Hung CF (2013). Dose-dense chemotherapy improves mechanisms of antitumor immune response. Cancer research.

[40] Peng S, Monie A, Kang TH, Hung CF, Roden R, Wu TC (2010). Efficient delivery of DNA vaccines using human papillomavirus pseudovirions. Gene therapy.

[41] Abràmoff MD, Magalhães PJ, Ram SJ (2004). Image processing with ImageJ. Biophotonics international.

[42] Lin KY, Lu D, Hung C-F, Peng S, Huang L, Jie C, Murillo F, Rowley J, Tsai YC, He L (2007). Ectopic expression of vascular cell adhesion molecule-1 as a new mechanism for tumor immune evasion. Cancer research.

[43] Chen S, Do JT, Zhang Q, Yao S, Yan F, Peters EC, Scholer HR, Schultz PG, Ding S (2006). Self-renewal of embryonic stem cells by a small molecule. Proc Natl Acad Sci USA.

