# Supplementary material for: RPN13/ADRM1 inhibitor reverses immunosuppression by myeloid-derived suppressor cells
Source: Oncotarget. 2016 Sep 17;7(42):68489–502. doi: 10.18632/oncotarget.12095 (PMC5340091; doi:10.18632/oncotarget.12095)
Supplement: Supplementary file 1 [file oncotarget-07-68489-s001.pdf]

RPN13/ADRM1 inhibitor reverses immunosuppression by myeloid-derived suppressor cells

Supplementary Materials

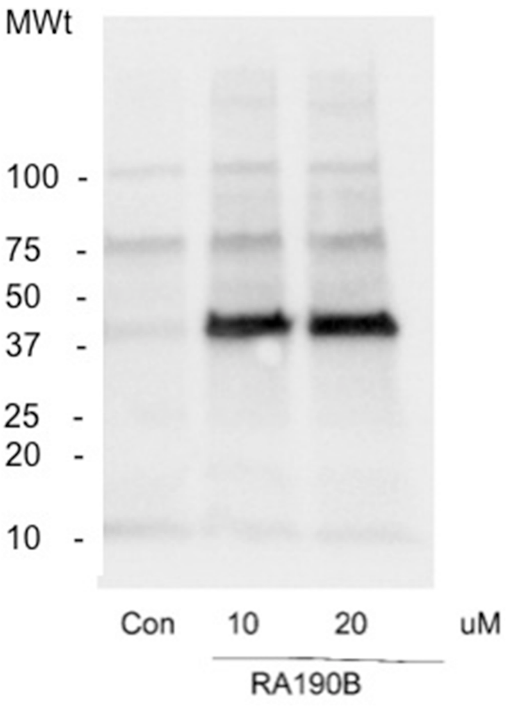

Supplementary Figure S1: RA190 binds to RPN13 in MDSCs. MDSC cell lysate was labeled with 10 or 20 µM of biotinylated RA190 (RA190B) for 45 minutes at 4°C. After labeling, equal amount of samples were boiled in Laemmli buffer, separated by SDS-PAGE, transferred to a PVDF membrane and probed with HRP-streptavidin for the recognition of biotinylated proteins.

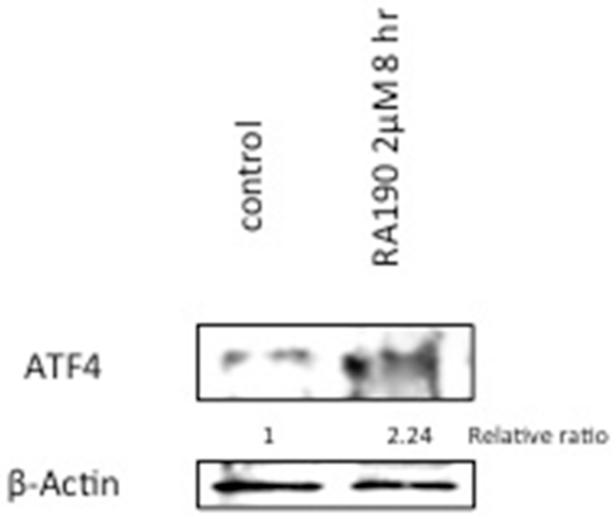

Supplementary Figure S2: Impact of RA190 treatment on ATF4 levels in MDSCs *in vitro*. Immunoblot of ATF4 expression level of MDSCs treated with PBS or RA190 (2 μM) for 8 hours.

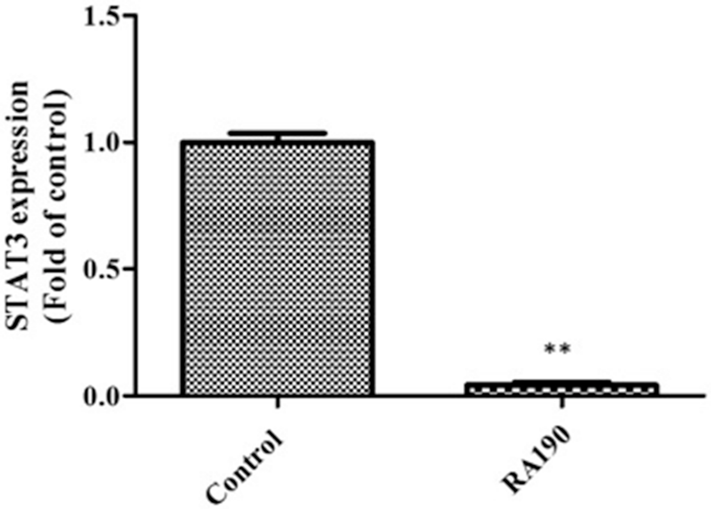

Supplementary Figure S3: Impact of RA190 treatment on Stat3 mRNA level in MDSCs *in vitro*. The MDSC cells were isolated from the spleen of tumor bearing mice, then isolated by Myeloid-Derived Suppressor Cell Isolation Kit. The 10^7 MDSC cells were treated with 1 μM RA190 for 24 hr. mRNA levels were analyzed by q-RT-PCR and normalized to GAPDH expression. Values are shown as mean ± SD (***P* < 0.01).

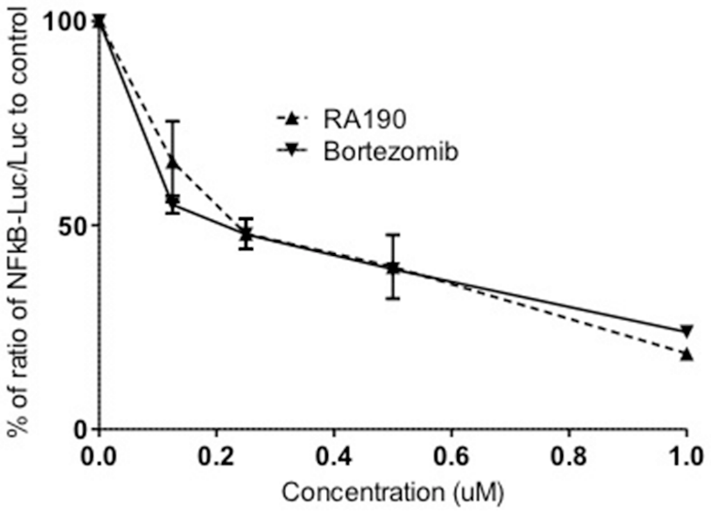

Supplementary Figure S4: RA190 inhibits the TNFα dependent NF-kB activation. 293 cells transiently transfected with a luciferase reporter construct driven by either an NFκB-dependent promotor (NFκB/FL) or a constitutive promoter (Luc) were treated with the compounds indicated and TNFα for 7 h. Upon the addition of luciferin, bioluminescence was measured in cell lysates using a luminometer. The % activation of the NFκB-dependent promoter (normalized by the constitutive reporter construct) as compared to TNFα stimulation in the absence of compound is presented. Values are shown as mean ± SD.

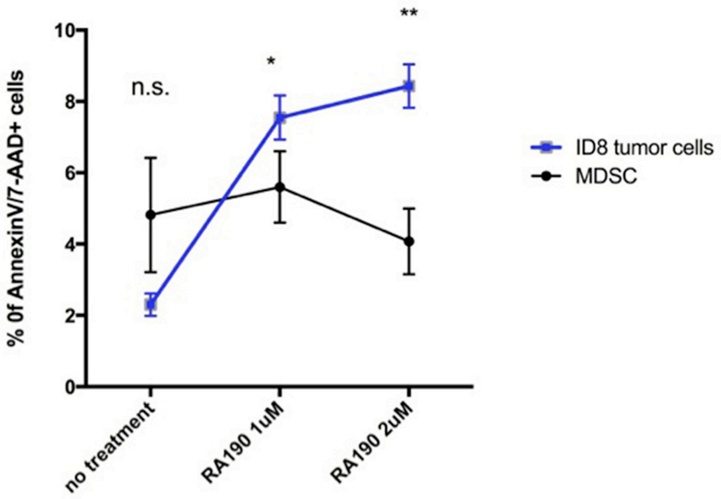

Supplementary Figure S5: RA190, in therapeutic dose, promotes apoptosis in tumor cells but not MDSC *in vitro*. MDSC and ID8 murine ovarian cancer cell line were treated with different concentrations of RA190 for 4 hours, stained for AnnexinV and 7-AAD, and analyzed by flow cytomery. Figure depicts the line graph showing the change in % of AnnexinV$^+$7-AAD$^+$ MDSCs and ID8 tumor cells when treated with different concentration of RA190. Values are shown as mean ± SD (*P < 0.05, **P < 0.01, n.s., not significant).
